# Supplementary material for: A Likelihood Approach for Real-Time Calibration of Stochastic Compartmental Epidemic Models
Source: PLoS Comput Biol. 2017 Jan 17;13(1):e1005257. doi: 10.1371/journal.pcbi.1005257 (PMC5240920; doi:10.1371/journal.pcbi.1005257)
Supplement: S1 File — (TAR.GZ) [file pcbi.1005257.s014.tar.gz › HSPH_Online-SI-Revision/EnKF07/n1-mild/n1-mild_EnKF2_table0.pdf]

1	newly 3 weeks spec	14.	9.22	{0., 18.}	112.714	1.35081	newly 3 weeks cumul	75.	92.99	{10., 383.}	121.453	0.502196	newly 5 weeks spec	14.	3.79	{0., 6.}	99.9286	1.63613	newly 5 weeks cumul	112.	102.18	{11., 392.}	107.857	0.60272	newly 55 weeks cumul	214.	111.27	{11., 396.}	92.257	0.788813
2	newly 3 weeks spec	8.	7.25	{0., 21.}	129.875	1.3096	newly 3 weeks cumul	39.	113.9	{6., 350.}	241.128	0.482831	newly 5 weeks spec	6.	2.	{0., 5.}	103.667	1.47668	newly 5 weeks cumul	52.	119.5	{6., 361.}	191.231	0.496155	newly 51 weeks cumul	74.	124.86	{6., 361.}	150.162	0.552358
3	newly 3 weeks spec	19.	23.07	{0., 79.}	137.737	0.925782	newly 3 weeks cumul	66.	206.35	{33., 568.}	238.318	0.389735	newly 5 weeks spec	13.	12.1	{0., 28.}	149.231	1.46933	newly 5 weeks cumul	88.	234.88	{39., 720.}	206.136	0.399232	newly 60 weeks cumul	152.	263.02	{45., 918.}	146.053	0.465754
4	newly 3 weeks spec	7.	28.73	{3., 51.}	340.143	0.451454	newly 3 weeks cumul	27.	127.75	{21., 237.}	382.704	0.471007	newly 5 weeks spec	12.	11.19	{0., 28.}	110.417	0.824218	newly 5 weeks cumul	48.	157.09	{27., 295.}	245.271	0.361843	newly 54 weeks cumul	336.	178.15	{29., 383.}	82.4137	0.636325
5	newly 3 weeks spec	21.	10.69	{0., 15.}	104.333	1.1182	newly 3 weeks cumul	87.	91.51	{6., 132.}	131.299	0.673611	newly 5 weeks spec	13.	4.62	{0., 6.}	108.	1.53296	newly 5 weeks cumul	118.	103.19	{6., 149.}	120.856	0.729711	newly 56 weeks cumul	175.	112.04	{8., 192.}	109.52	0.841414
6	newly 3 weeks spec	25.	24.43	{0., 45.}	124.28	0.903514	newly 3 weeks cumul	80.	168.65	{18., 582.}	169.863	0.415955	newly 5 weeks spec	28.	10.58	{0., 43.}	104.143	1.6105	newly 5 weeks cumul	143.	195.29	{18., 695.}	127.643	0.519105	newly 60 weeks cumul	316.	216.17	{18., 880.}	94.0158	0.715808
7	newly 3 weeks spec	43.	20.37	{0., 38.}	96.3953	1.07293	newly 3 weeks cumul	128.	142.45	{15., 453.}	116.789	0.55726	newly 5 weeks spec	19.	8.92	{0., 16.}	106.947	1.34194	newly 5 weeks cumul	180.	164.52	{17., 658.}	108.422	0.627701	newly 59 weeks cumul	287.	189.16	{18., 798.}	99.6098	0.75558
8	newly 3 weeks spec	17.	6.82	{0., 18.}	88.9412	1.18785	newly 3 weeks cumul	45.	70.34	{6., 93.}	130.222	0.412273	newly 5 weeks spec	19.	1.82	{0., 5.}	93.2632	1.78689	newly 5 weeks cumul	80.	75.53	{6., 137.}	100.938	0.54271	newly 51 weeks cumul	385.	77.94	{6., 138.}	88.5766	1.09595
9	newly 3 weeks spec	39.	9.97	{0., 20.}	85.2051	1.32081	newly 3 weeks cumul	109.	125.68	{5., 387.}	119.927	0.606292	newly 5 weeks spec	28.	2.24	{0., 6.}	92.	1.72476	newly 5 weeks cumul	160.	132.08	{6., 417.}	101.588	0.67592	newly 50 weeks cumul	206.	135.17	{6., 435.}	93.1796	0.734861
10	newly 3 weeks spec	14.	11.24	{0., 16.}	103.714	0.922366	newly 3 weeks cumul	60.	82.82	{8., 99.}	121.567	0.459772	newly 5 weeks spec	11.	3.44	{0., 7.}	90.3636	1.21469	newly 5 weeks cumul	94.	92.08	{8., 112.}	103.957	0.55406	newly 51 weeks cumul	109.	96.75	{10., 116.}	100.083	0.584461
11	newly 3 weeks spec	16.	10.14	{0., 33.}	115.625	1.35025	newly 3 weeks cumul	57.	77.45	{3., 186.}	143.07	0.602496	newly 5 weeks spec	6.	4.4	{0., 16.}	129.	1.39777	newly 5 weeks cumul	78.	88.13	{3., 262.}	129.577	0.677856	newly 50 weeks cumul	387.	100.29	{3., 330.}	85.0775	1.15791
12	newly 3 weeks spec	18.	6.93	{0., 17.}	86.9444	1.2671	newly 3 weeks cumul	54.	73.01	{11., 88.}	103.537	0.334966	newly 5 weeks spec	20.	4.51	{0., 7.}	98.95	1.65038	newly 5 weeks cumul	85.	82.68	{12., 105.}	94.1882	0.444433	newly 51 weeks cumul	271.	96.24	{13., 168.}	86.9815	0.831788
13	newly 3 weeks spec	19.	18.24	{0., 36.}	118.211	0.79409	newly 3 weeks cumul	55.	100.73	{28., 215.}	113.836	0.258975	newly 5 weeks spec	8.	3.39	{0., 11.}	88.875	1.21598	newly 5 weeks cumul	79.	112.08	{30., 256.}	93.8734	0.283954	newly 52 weeks cumul	97.	117.42	{30., 266.}	86.2474	0.31555
14	newly 3 weeks spec	15.	14.91	{0., 24.}	135.4	1.05115	newly 3 weeks cumul	78.	97.77	{7., 138.}	145.397	0.61911	newly 5 weeks spec	10.	4.65	{0., 9.}	108.3	1.3816	newly 5 weeks cumul	112.	109.83	{8., 210.}	128.616	0.702112	newly 56 weeks cumul	131.	126.2	{8., 229.}	127.634	0.733964
15	newly 3 weeks spec	27.	8.28	{0., 16.}	87.3333	1.10803	newly 3 weeks cumul	78.	98.56	{7., 140.}	142.436	0.615903	newly 5 weeks spec	19.	2.83	{0., 6.}	94.2632	1.70055	newly 5 weeks cumul	124.	105.62	{7., 169.}	116.548	0.723019	newly 56 weeks cumul	280.	109.66	{7., 181.}	96.1857	0.985046
16	newly 3 weeks spec	11.	11.64	{0., 17.}	118.364	0.658748	newly 3 weeks cumul	35.	61.15	{14., 96.}	127.286	0.277784	newly 5 weeks spec	16.	4.7	{0., 8.}	93.75	1.31062	newly 5 weeks cumul	59.	73.86	{19., 118.}	112.644	0.385957	newly 54 weeks cumul	273.	82.37	{20., 152.}	88.685	0.878209
17	newly 3 weeks spec	20.	8.37	{0., 16.}	87.85	1.0637	newly 3 weeks cumul	50.	90.75	{5., 143.}	164.1	0.457478	newly 5 weeks spec	12.	4.22	{0., 10.}	97.1667	1.37019	newly 5 weeks cumul	78.	101.29	{5., 157.}	133.962	0.551573	newly 57 weeks cumul	269.	119.82	{5., 236.}	91.9628	0.896051
18	newly 3 weeks spec	14.	6.97	{0., 14.}	112.071	1.44627	newly 3 weeks cumul	53.	80.79	{3., 72.}	169.189	0.63669	newly 5 weeks spec	18.	1.69	{0., 1.}	98.8333	2.04217	newly 5 weeks cumul	81.	85.43	{3., 93.}	140.16	0.767548	newly 50 weeks cumul	239.	88.52	{3., 93.}	102.527	1.14744
19	newly 3 weeks spec	24.	8.31	{0., 12.}	98.2917	1.34418	newly 3 weeks cumul	86.	67.07	{5., 71.}	101.872	0.644492	newly 5 weeks spec	24.	4.35	{0., 7.}	98.9583	1.70323	newly 5 weeks cumul	128.	77.98	{5., 80.}	98.5781	0.743826	newly 57 weeks cumul	218.	87.7	{5., 107.}	93.5596	0.904098
20	newly 3 weeks spec	15.	9.68	{0., 13.}	101.333	0.945541	newly 3 weeks cumul	66.	69.98	{6., 135.}	103.727	0.502366	newly 5 weeks spec	18.	4.24	{0., 8.}	93.	1.47878	newly 5 weeks cumul	102.	80.8	{6., 162.}	96.0196	0.606047	newly 54 weeks cumul	153.	93.47	{6., 322.}	92.2941	0.719171
21	newly 3 weeks spec	19.	12.24	{0., 38.}	117.789	1.39809	newly 3 weeks cumul	59.	66.79	{5., 134.}	117.949	0.5542	newly 5 weeks spec	16.	6.36	{0., 7.}	113.375	1.66903	newly 5 weeks cumul	93.	81.43	{5., 212.}	111.882	0.683676	newly 54 weeks cumul	145.	92.	{5., 288.}	102.855	0.823485
22	newly 3 weeks spec	17.	13.94	{0., 36.}	115.176	1.10603	newly 3 weeks cumul	45.	102.52	{4., 231.}	213.867	0.538384	newly 5 weeks spec	12.	5.98	{0., 11.}	108.5	1.41739	newly 5 weeks cumul	68.	116.11	{4., 307.}	172.809	0.621852	newly 56 weeks cumul	388.	140.23	{5., 409.}	89.4871	1.10286
23	newly 3 weeks spec	18.	9.08	{0., 19.}	76.4444	0.800149	newly 3 weeks cumul	69.	98.63	{11., 217.}	120.652	0.414874	newly 5 weeks spec	27.	2.47	{0., 5.}	90.8519	1.63484	newly 5 weeks cumul	118.	105.18	{12., 230.}	96.	0.521119	newly 51 weeks cumul	241.	110.79	{12., 262.}	84.8506	0.731938
24	newly 3 weeks spec	25.	14.43	{0., 10.}	130.28	1.76327	newly 3 weeks cumul	83.	82.63	{3., 95.}	114.494	0.686138	newly 5 weeks spec	20.	5.26	{0., 3.}	109.1	1.98544	newly 5 weeks cumul	121.	97.99	{3., 96.}	113.992	0.810193	newly 53 weeks cumul	298.	104.66	{3., 101.}	97.1275	1.13135
25	newly 3 weeks spec	12.	20.05	{1., 40.}	163.75	0.572585	newly 3 weeks cumul	34.	82.98	{10., 140.}	178.824	0.293127	newly 5 weeks spec	11.	10.63	{0., 9.}	137.364	1.00004	newly 5 weeks cumul	59.	107.66	{13., 200.}	149.525	0.347769	newly 50 weeks cumul	239.	130.94	{17., 276.}	101.456	0.786736
26	newly 3 weeks spec	22.	14.5	{0., 30.}	113.909	1.33303	newly 3 weeks cumul	61.	113.7	{5., 303.}	188.557	0.633326	newly 5 weeks spec	13.	5.45	{0., 12.}	103.769	1.4502	newly 5 weeks cumul	91.	128.01	{5., 325.}	157.791	0.719833	newly 53 weeks cumul	122.	145.72	{5., 772.}	142.656	0.77331
27	newly 3 weeks spec	11.	12.81	{0., 36.}	155.909	1.16963	newly 3 weeks cumul	44.	76.57	{4., 127.}	163.159	0.518696	newly 5 weeks spec	10.	5.25	{0., 12.}	118.9	1.59434	newly 5 weeks cumul	72.	89.58	{4., 259.}	139.75	0.652586	newly 50 weeks cumul	169.	108.38	{4., 366.}	110.189	0.916641
28	newly 3 weeks spec	15.	10.55	{0., 36.}	120.2	1.36206	newly 3 weeks cumul	48.	79.09	{4., 171.}	153.229	0.551322	newly 5 weeks spec	16.	5.35	{0., 5.}	107.188	1.7713	newly 5 weeks cumul	77.	92.18	{4., 262.}	129.377	0.662134	newly 56 weeks cumul	293.	111.52	{4., 398.}	91.7338	1.06898
29	newly 3 weeks spec	19.	16.6	{0., 79.}	148.	1.59368	newly 3 weeks cumul	60.	110.43	{4., 381.}	177.483	0.555617	newly 5 weeks spec	14.	3.74	{0., 3.}	110.143	1.89034	newly 5 weeks cumul	96.	120.57	{4., 456.}	147.948	0.704821	newly 53 weeks cumul	143.	134.24	{4., 632.}	131.846	0.835961
30	newly 3 weeks spec	28.	7.54	{0., 12.}	94.0714	1.42648	newly 3 weeks cumul	82.	87.13	{6., 65.}	124.744	0.588285	newly 5 weeks spec	11.	3.46	{0., 4.}	107.273	1.57512	newly 5 weeks cumul	114.	94.67	{6., 75.}	115.728	0.684527	newly 57 weeks cumul	214.	108.19	{6., 112.}	104.173	0.889535
31	newly 3 weeks spec	34.	25.55	{0., 86.}	96.9118	0.898729	newly 3 weeks cumul	96.	175.9	{12., 459.}	145.917	0.447085	newly 5 weeks spec	13.	7.61	{0., 21.}	95.7692	1.12304	newly 5 weeks cumul	127.	196.78	{14., 551.}	128.22	0.47497	newly 55 weeks cumul	143.	208.29	{14., 636.}	122.301	0.485403
32	newly 3 weeks spec	11.	16.48	{0., 52.}	182.545	1.23235	newly 3 weeks cumul	53.	139.94	{4., 573.}	232.566	0.519548	newly 5 weeks spec	12.	3.12	{0., 7.}	99.5	1.67216	newly 5 weeks cumul	70.	149.2	{4., 611.}	198.714	0.578018	newly 53 weeks cumul	339.	154.7	{4., 671.}	96.0354	1.03136
33	newly 3 weeks spec	38.	10.78	{0., 16.}	89.3684	1.1007	newly 3 weeks cumul	103.	87.84	{14., 277.}	110.835	0.620644	newly 5 weeks spec	18.	3.66	{0., 7.}	92.8889	1.43851	newly 5 weeks cumul	138.	96.98	{14., 299.}	103.029	0.674032	newly 54 weeks cumul	205.	107.72	{16., 389.}	95.8829	0.77139
34	newly 3 weeks spec	13.	27.11	{0., 93.}	237.308	0.971248	newly 3 weeks cumul	28.	100.45	{5., 324.}	311.179	0.463642	newly 5 weeks spec	11.	11.48	{0., 42.}	154.364	1.3163	newly 5 weeks cumul	49.	130.38	{7., 504.}	247.918	0.543658	newly 53 weeks cumul	176.	145.6	{7., 807.}	122.693	0.862352
35	newly 3 weeks spec	36.	9.56	{0., 18.}	94.5	1.57966	newly 3 weeks cumul	89.	113.38	{8., 403.}	130.09	0.577408	newly 5 weeks spec	34.	3.01	{0., 5.}	97.1471	1.97904	newly 5 weeks cumul	162.	121.77	{8., 464.}	103.747	0.729248	newly 55 weeks cumul	281.	127.74	{8., 464.}	90.3986	0.885518
36	newly 3 weeks spec	29.	14.62	{2., 17.}	92.9655	0.76665	newly 3 weeks cumul	101.	84.42	{13., 106.}	100.693	0.539121	newly 5 weeks spec	18.	6.3	{0., 8.}	100.778	1.26875	newly 5 weeks cumul	133.	99.82	{17., 151.}	98.406	0.571682	newly 57 weeks cumul	143.	115.82	{17., 171.}	98.965	0.551603
37	newly 3 weeks spec	16.	27.61	{0., 116.}	187.563	0.862549	newly 3 weeks cumul	67.	140.2	{7., 534.}	185.284	0.495239	newly 5 weeks spec	19.	9.01	{0., 18.}	108.263	1.33534	newly 5 weeks cumul	103.	164.62	{7., 712.}	159.34	0.587452	newly 53 weeks cumul	179.	177.48	{8., 829.}	125.531	0.739713
38	newly 3 weeks spec	31.	6.77	{0., 18.}	83.8387	1.37747	newly 3 weeks cumul	127.	107.25	{10., 118.}	113.11	0.674333	newly 5 weeks spec	22.	2.28	{0., 7.}	91.6364	1.71083	newly 5 weeks cumul	168.	113.16	{10., 137.}	103.821	0.735709	newly 57 weeks cumul	211.	120.91	{10., 192.}	96.6019	0.7795
39	newly 3 weeks spec	16.	14.17	{0., 26.}	125.813	1.18254	newly 3 weeks cumul	52.	80.11	{8., 193.}	143.519	0.492592	newly 5 weeks spec	14.	7.21	{0., 9.}	107.929	1.35778	newly 5 weeks cumul	79.	97.33	{11., 202.}	129.557	0.570741	newly 55 weeks cumul	143.	115.57	{11., 242.}	110.371	0.718054
40	newly 3 weeks spec	28.	7.7	{0., 15.}	96.	1.49385	newly 3 weeks cumul	115.	91.76	{9., 209.}	93.7739	0.588265	newly 5 weeks spec	12.	2.87	{0., 5.}	102.083	1.66051	newly 5 weeks cumul	156.	99.4	{11., 243.}	90.2308	0.662637	newly 58 weeks cumul	187.	104.37	{11., 247.}	88.4439	0.710968
41	newly 3 weeks spec	19.	14.35	{0., 28.}	111.947	1.00785	newly 3 weeks cumul	73.	108.46	{8., 198.}	133.479	0.512483	newly 5 weeks spec	10.	3.79	{0., 12.}	96.5	1.31314	newly 5 weeks cumul	107.	119.66	{8., 248.}	114.505	0.588762	newly 59 weeks cumul	145.	124.92	{9., 283.}	104.248	0.668117
42	newly 3 weeks spec	12.	9.64	{0., 13.}	124.167	1.18866	newly 3 weeks cumul	104.	82.8	{6., 88.}	101.923	0.66061	newly 5 weeks spec	0.1	3.35	{0., 5.}	3396.	0.468585	newly 5 weeks cumul	106.	91.55	{6., 88.}	105.142	0.645612	newly 58 weeks cumul	106.	95.8	{6., 96.}	105.811	0.632743
43	newly 3 weeks spec	28.	14.76	{0., 22.}	103.786	1.26568	newly 3 weeks cumul	117.	101.94	{10., 271.}	86.5983	0.508776	newly 5 weeks spec	11.	8.4	{0., 17.}	136.182	1.43155	newly 5 weeks cumul	151.	122.19	{10., 346.}	90.7881	0.576986	newly 60 weeks cumul	167.	142.65	{10., 460.}	98.3293	0.607752
44	newly 3 weeks spec	20.	11.57	{0., 12.}	115.05	1.25458	newly 3 weeks cumul	78.	56.55	{5., 47.}	104.962	0.693917	newly 5 weeks spec	6.	5.34	{0., 7.}	143.	1.28124	newly 5 weeks cumul	92.	69.49	{5., 56.}	110.098	0.71934	newly 52 weeks cumul	103.	79.06	{7., 70.}	111.748	0.734641
45	newly 3 weeks spec	25.	9.97	{0., 17.}	94.44	1.2419	newly 3 weeks cumul	80.	85.67	{6., 92.}	100.438	0.485323	newly 5 weeks spec	16.	3.94	{0., 7.}	95.125	1.526	newly 5 weeks cumul	114.	95.32	{6., 98.}	95.7544	0.574764	newly 50 weeks cumul	184.	105.18	{7., 229.}	90.1848	0.719745
46	newly 3 weeks spec	21.	8.98	{0., 12.}	94.6667	1.1544	newly 3 weeks cumul	65.	83.7	{7., 168.}	128.738	0.514843	newly 5 weeks spec	19.	4.55	{0., 7.}	97.	1.53744	newly 5 weeks cumul	90.	94.37	{8., 168.}	116.744	0.575014	newly 57 weeks cumul	370.	110.82	{8., 189.}	88.8757	1.00288
47	newly 3 weeks spec	10.	10.51	{0., 29.}	128.5	1.03908	newly 3 weeks cumul	30.	107.75	{8., 434.}	298.9	0.411188	newly 5 weeks spec	5.	2.98	{0., 6.}	111.2	1.19258	newly 5 weeks cumul	45.	115.41	{8., 478.}	216.644	0.431276	newly 55 weeks cumul	300.	121.68	{8., 478.}	86.7133	0.89588
48	newly 3 weeks spec	18.	23.51	{0., 81.}	143.833	0.897481	newly 3 weeks cumul	78.	154.63	{13., 381.}	175.346	0.446601	newly 5 weeks spec	4.	7.57	{0., 15.}	193.75	0.86046	newly 5 weeks cumul	93.	174.92	{13., 661.}	168.172	0.466863	newly 50 weeks cumul	172.	188.91	{13., 838.}	120.808	0.605993
49	newly 3 weeks spec	6.	7.81	{0., 13.}	136.833	0.755396	newly 3 weeks cumul	35.	63.94	{6., 83.}	144.743	0.404891	newly 5 weeks spec	12.	2.74	{0., 6.}	96.6667	1.54501	newly 5 weeks cumul	58.	70.87	{6., 175.}	114.224	0.502513	newly 54 weeks cumul	236.	83.37	{6., 177.}	90.7585	0.959575
50	newly 3 weeks spec	20.	8.99	{0., 12.}	114.25	1.64876	newly 3 weeks cumul	56.	86.2	{7., 116.}	143.893	0.466228	newly 5 weeks spec	23.	3.22	{0., 5.}	100.261	1.98369	newly 5 weeks cumul	95.	94.07	{7., 121.}	118.179	0.611562	newly 51 weeks cumul	220.	102.39	{7., 205.}	96.8773	0.88298
